# Therapeutic angiogenesis by transplantation of induced pluripotent stem cell-derived Flk-1 positive cells

**DOI:** 10.1186/1471-2121-11-72

**Published:** 2010-09-22

**Authors:** Hirohiko Suzuki, Rei Shibata, Tetsutaro Kito, Masakazu Ishii, Ping Li, Toru Yoshikai, Naomi Nishio, Sachiko Ito, Yasushi Numaguchi, Jun K Yamashita, Toyoaki Murohara, Kenichi Isobe

**Affiliations:** 1 Department of Cardiology, Nagoya University Graduate School of Medicine, 65 Tsurumai-cho, Showa-ku, Nagoya, Aichi 466-8560, Japan; 2 Department of Immunology, Nagoya University Graduate School of Medicine, 65 Tsurumai-cho, Showa-ku, Nagoya, Aichi 466-8560, Japan; 3 Laboratory of Stem Cell Differentiation, Stem Cell Research Center, Institute for Frontier Medical Sciences, Kyoto University, 53 Shogoin Kawahara-cho, Sakyo-ku, Kyoto 606-8507, Japan

## Abstract

**Background:**

Induced pluripotent stem (iPS) cells are the novel stem cell population induced from somatic cells. It is anticipated that iPS will be used in the expanding field of regenerative medicine. Here, we investigated whether implantation of fetal liver kinase-1 positive (Flk-1^+^) cells derived from iPS cells could improve angiogenesis in a mouse hind limb model of ischemia.

**Results:**

Flk-1^+ ^cells were induced from iPS cells after four to five days of culture. Hind limb ischemia was surgically induced and sorted Flk-1^+ ^cells were directly injected into ischemic hind limbs of athymic nude mice. Revascularization of the ischemic hind limb was accelerated in mice that were transplanted with Flk-1^+ ^cells compared with control mice, which were transplanted with vehicle, as evaluated by laser Doppler blood flowmetry. Transplantation of Flk-1^+ ^cells also increased expression of VEGF mRNA in ischemic tissue compared to controls.

**Conclusions:**

Direct local implantation of iPS cell-derived Flk-1^+ ^cells would salvage tissues from ischemia. These data indicate that iPS cells could be valuable in the therapeutic induction of angiogenesis.

## Background

There are increasing numbers of patients around the world with peripheral arterial disease (PAD) [1]. Promotion of collateral vessel formation and angiogenesis in such patients is an important therapeutic strategy to minimize tissue injury associated with severe ischemia. Circulating endothelial progenitor cells (EPCs) have been discovered in peripheral blood and shown to participate in postnatal neovascularization after mobilization from bone marrow (BM) [2,3]. Based upon those discoveries, we conducted therapeutic angiogenesis using autologous BM-derived mononuclear cell (BM-MNC) implantation (the TACT trial) into the ischemic muscles in patients with critical limb ischemia [4-6]. However, patients with very severe PAD undergoing chronic hemodialysis or uncontrolled diabetes had poor responses to the TACT procedure [5]. Moreover, recent data indicate that patients with severe ischemic heart disease and/or multiple coronary risk factors have a reduced number of circulating EPCs, diminished angiogenic function of their EPCs and a poor response to angiogenic cell therapy [7-9]. Therefore, it is necessary to discover an alternative source of stem/progenitor cells for therapeutic angiogenesis.

Recently, novel embryonic stem (ES) cell-like pluripotent stem cells were generated from mouse skin fibroblasts by introduction of four transcriptional factors (Oct3/4, Sox2, Klf4, c-Myc)[10]. Termed induced pluripotent stem (iPS) cells, they could be used repetitively and were capable of differentiating into various kinds of cells as needed[11-15]. Recently, it was reported that various cardiovascular cells could be directionally induced from mouse and human iPS cell-derived fetal liver kinase-1 positive (Flk-1^+^) cells *in vitro *[11,16]. Thus, iPS cells open new possibilities for cell-based regenerative medicine that will circumvent the ethical controversies and immune-related problems associated with ES cells. Here, we investigated whether implantation of iPS-derived Flk-1^+ ^cells could augment the process of ischemia-induced angiogenesis *in vivo*.

## Results

### Differentiation of iPS cells to Flk-1^+ ^cells

**Figure 1:**
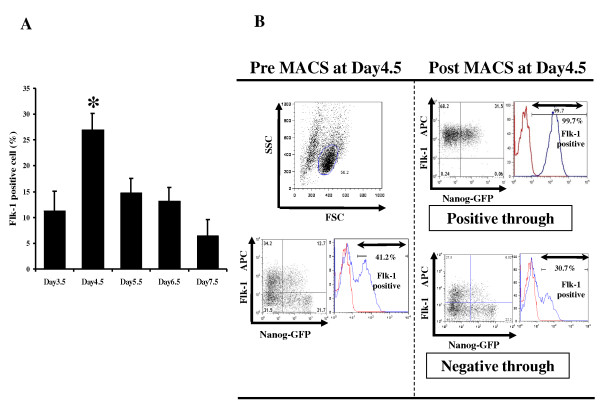
**Purification of Flk-1+ cells from iPS cells**. A) Flk-1 expression profiles from 3.5 days to 7.5 days of cultivation as determined by flow cytometric analysis. (* p < 0.05 Day4.5 vs Day3.5, 5.5, 6.5 and 7.5). B) FACS analysis of pre and post MACS-sorted Flk-1^+ ^cells at day 4.5. More than 99% of enriched cells were positive for Flk-1. Some of these purified Flk-1^+ ^cells were positive for Nanog-GFP.

Undifferentiated iPS cells were cultured on collagen IV-coated dishes with DM as described previously [11]. Firstly, we assessed the time course of Flk-1^+ ^cell appearance by fluorescence-activated cell sorter (FACS). Flk-1^+ ^cells appeared after 3.5 days of culture and peaked on day 4.5 (Figure 1A). The average frequencies of Flk-1^+ ^cells were 11.3% (day 3.5), 27% (day 4.5), 14.9% (day 5.5), 13.2% (day 6.5) and 6.5% (day 7.5), confirming a previous report [11]. Based on these findings, we sorted Flk-1^+ ^cells by magnetic-activated cell sorting (MACS) at day 4.5 of differentiation in the present study. FACS analysis of MACS-sorted positive through cells showed that more than 99% of these cells were positive for Flk-1 (Figure 1B). We also found that MACS-purified Flk-1 positive cells were sorted in not only Nanog-GFP positive population but also Nanog-GFP negative cell population (Figure 1B).

**Figure 2:**
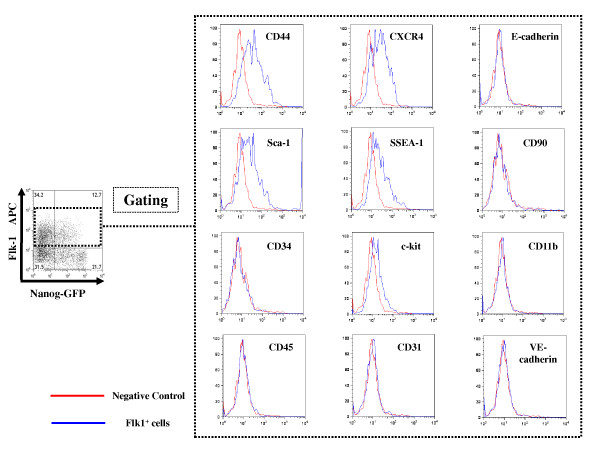
**Characterization of Flk1+ cells**. Differentiated iPS cells were analyzed by FACS. Differentiated iPS cells were stained with Flk1-APC and additional cell marker (c-kit, Sca-1, CD11b, CD31, VECAD, CD34, CD44, CD45, CD90, SSEA-1, CXCR4 and ECAD). Additional cell marker was analyzed after gating Flk1^+ ^cells. Red line shows the result of the negative control and Blue line shows the result of the sample stained by each antibody.

We also tried to characterize the Flk1^+ ^cells by FACS analysis. Gated Flk1^+ ^cells showed CD11b negative, CD45 negative, CD31 negative, VE-cadherin negative, E-cadherin negative, CD34 negative or CD90 negative (Figure 2). CD44 positive cells accounted for 39.4% of gated Flk1+ cells, CXCR4 positive cells for 29.1%, Sca-1 positive cells for 24.8%, SSEA-1 positive cells for 23.5% and c-kit positive cells for 8.2%. Flk1^+ ^cells are considered as relatively immature and still have an aspect as heterogeneous stem cells at the upstream position.

**Figure 3:**
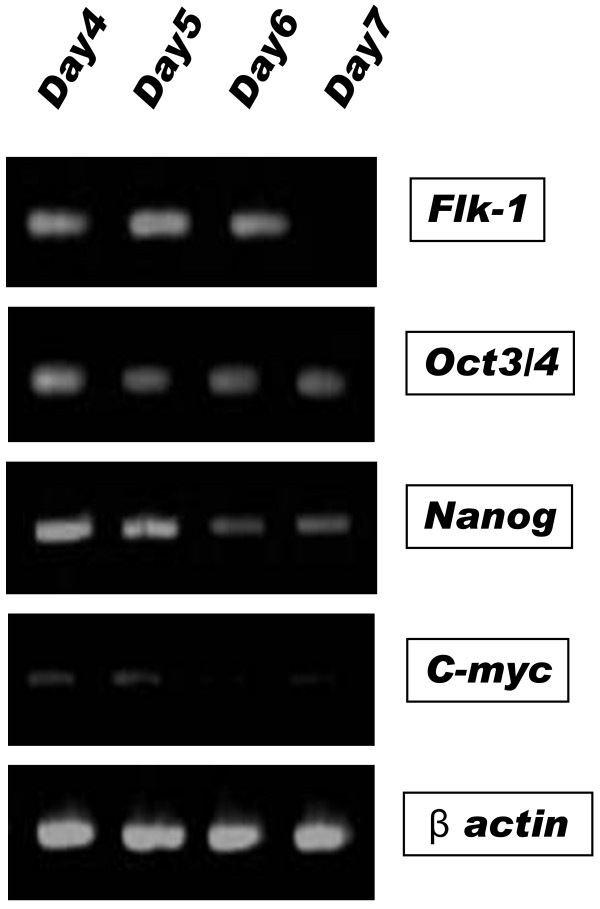
**Time course RT-CPR in vitro**. The expression of Flk-1 was peaked at Day5 from differentiation. Undifferentiated iPS cells markers, Nanog and Oct3/4, were strongly expressed in early phase and started to gradually decrease with differentiation. We also observed the transient expression of c-myc at Day4 and Day5.

We confirmed the expression of undifferentiated markers such as Nanog and Oct3/4 on differentiation at day 4, 5, 6 and 7 by RT-PCR (Figure 3). Undifferentiated iPS cells markers, Nanog and Oct3/4, were strongly expressed in early phase and started to gradually decrease with differentiation. We also observed the transient expression of c-myc.

### Augmentation of ischemia-induced angiogenesis by Flk-1^+ ^cells derived from iPS cells

**Figure 4:**
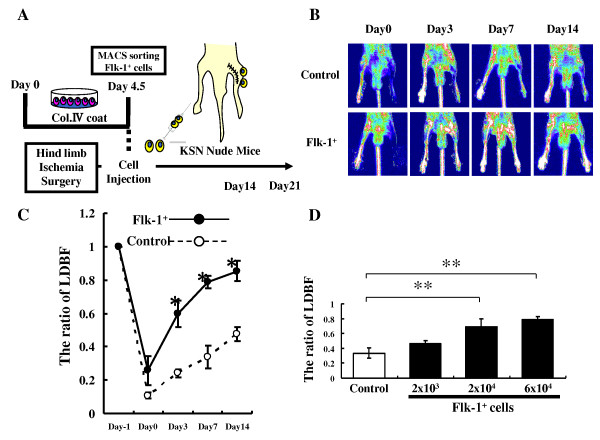
**Effects of cell transplantation on blood flow recovery in the ischemic hind limb**. A) Strategy to purify Flk-1^+ ^cells and to transplant them into ischemic hind limb tissues. B) Representative LDBF images. A low perfusion signal (dark blue) was observed in the ischemic left hind limb of control mice, whereas high perfusion signals (white to red) were detected in Flk-1^+ ^cell-transplanted animals (6 × 10^4 ^cells) on postoperative days 3, 7 and 14. C) Quantitative analysis of the ischemic/nonischemic limb LDBF ratio on pre (Day-1) and post operative days 0, 3, 7 and 14 (n = 4). *p < 0.05 Flk1^+ ^cells (6×10^4^) injected mice vs. control mice. D) Cell dose-dependent effect of transplantation seven days after surgery. Flk-1^+ ^cells (2 × 10^3^, 2 × 10^4 ^or 6 × 10^4 ^cells) or PBS as a control were injected into the ischemic limb at postoperative day 1 (n = 4/each group, **p < 0.05 2×10^4 ^or 6×10^4 ^vs. control mice).

We examined whether in vivo implantation of Flk-1^+ ^cells derived from iPS cells could augment ischemia-induced angiogenesis using a mouse model of hind limb ischemia. KSN athymic nude mice underwent surgical induction of unilateral hind limb ischemia. Sorted Flk-1^+ ^cells (6×10^4^cells/mouse) or control PBS were injected into adductor muscles in the ischemic limb at postoperative day 1 (Figure 4A). All mice survived from surgery and appeared healthy during the follow-up period. Body weight and blood pressure did not differ among the groups. We show the representative LDBF images of hind limb blood flow of recipient mice just after the surgery and at the different time points after surgery (Figure 4B). In control mice, hind limb perfusion fell precipitously after surgery, increased to 20 - 30% of the nonischemic limb by day 3, and increased to 40-50% of the nonischemic limb by day 14 (Figure 4C). However, mice transplanted with Flk-1^+ ^cells showed significant increases in hind limb blood relative to control at 3, 7 and 14 days after hind limb surgery (Figure 4B,C). We next assessed dose-dependency of cell transplantation at 7 days after surgery. Smaller doses of cells (2 × 10^4 ^cells/mouse) also yielded improvements in blood perfusion (Figure 4D). However, mice treated with 2 × 10^3 ^cells did not achieve a significant improvement in blood perfusion in the ischemic limb compared to control mice (Figure 4D).

### Implanted Flk-1^+ ^cells augmented the expression of VEGF in ischemic tissues

**Figure 5:**
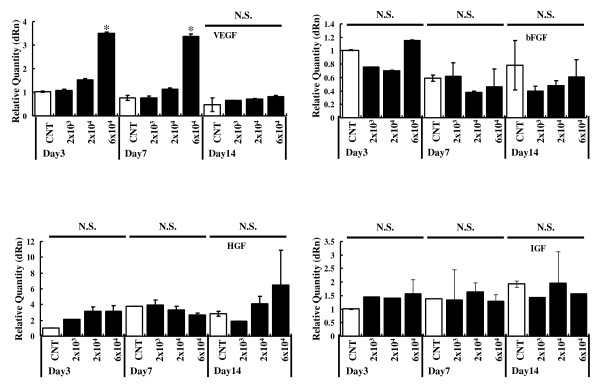
**Flk-1+ cell implantation stimulated VEGF, basic FGF, HGF and IGF expression in ischemic tissue**. VEGF, basic FGF, HGF and IGF synthesis in ischemic hind limb muscles was determined by real-time RT-PCR following transplantation of the Flk-1^+ ^cells (2 × 10^3^, 2 × 10^4 ^or 6 × 10^4 ^cells) or PBS-injection as the control(CNT). Results are expressed as the level of VEGF, basic FGF, HGF and IGF mRNA to day three control. GAPDH mRNA levels were used as the internal control. *P < 0.05 6×10^4 ^vs. CNT at each day (n = 4 in each groups). N.S. = not significant difference between groups at same day.

We investigated whether implantation of Flk-1^+ ^cells would up-regulate VEGF mRNA expression in ischemic hind limb tissues in chronological order. At postoperative days 3 and 7, VEGF mRNA expression increased significantly in mice transplanted with Flk-1^+ ^cells (6 × 10^4 ^cells) compared to that of control mice (Figure 5). Smaller doses of cells (2 × 10^4 ^cells/mouse) also slightly increased VEGF mRNA expression. At day 14, there was no significant difference between the groups. However, the dose-dependency for expression of VEGF mRNA was apparent.

We next examined the synthesis of basic FGF, HGF and IGF in ischemic muscle after cell transplantation by real-time PCR. There were no significant difference in these mRNA levels between mice transplanted with Flk-1^+ ^cells and control mice (Figure 5).

### Tracing Flk-1^+ ^cells in vitro and at chronic phase in vivo

**Figure 6:**
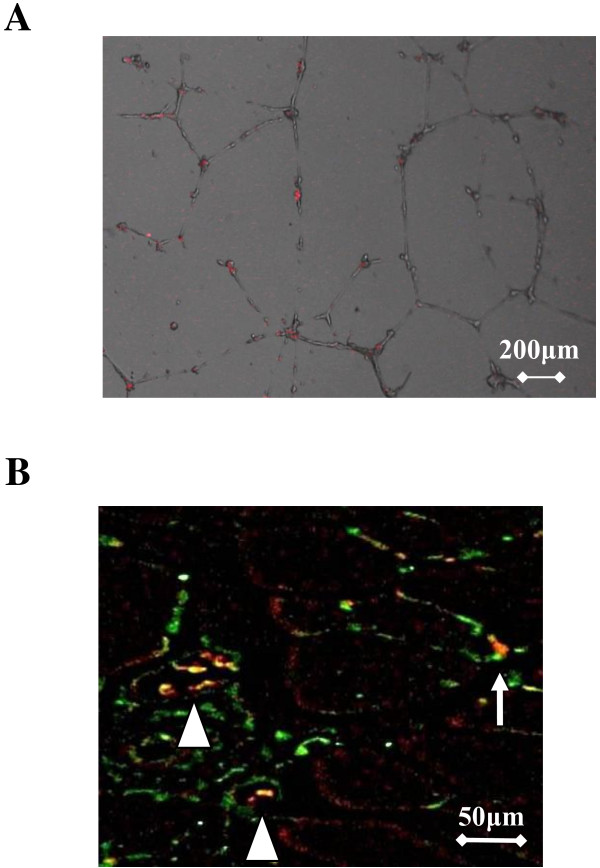
**Tracking Flk-1^+ ^cells in vitro and at chronic phase in vivo**. A) Tube formation assay in vitro. PKH26 red-labeled Flk-1^+ ^iPS cells were co-cultured with HUVECs for 24 hours on Matrigel. iPS cells (red) were confirmed to be incorporated into network structures. The bar indicates 200 μm. B) Double fluorescence staining of CD31 (green) and PKH26 (red) in ischemic adductor muscles on postoperative day 21. Co-localization is indicated by yellow in the merged images (magnification, ×400; bar indicates 50 μm). Flk-1^+ ^cells (6 × 10^4 ^cells) were stained with PKH26 red and then injected into ischemic adductor muscles. Double positive cells (▲) and single positive PKH26 cells(↑) are indicated.

We examined whether Flk-1^+ ^cells could be incorporated into the vascular network in vitro. Figure 6A shows representative photographs of PKH26-labeled Flk-1^+ ^iPS cells and HUVECs after 24 hours of co-culture on a Matrigel. Incorporation of Flk-1^+ ^iPS cells (red) into the network structures was confirmed.

Using PKH26-labeled Flk1^+ ^cells, we examined whether in vivo implanted Flk-1^+ ^cells survived and differentiated into vascular endothelial cells in ischemic tissues (Figure 6B). Transplanted PKH26-positive cells were found in the ischemic area even at postoperative day 21. Immunofluorescence histology revealed that some of the PKH26-positive cells were co-stained with the endothelial cell marker CD31 (Figure 6B). These results indicated that implanted Flk-1^+ ^cells contributed to vascular structure formation at postoperative day 21. We could not detect any tumors in the mice transplanted with Flk-1^+ ^(6 × 10^4^) cells through the 60 days after transplantation (n = 3).

## Discussion

The present study provides evidence that implantation of Flk-1^+ ^cells differentiated from mouse iPS cells promote angiogenesis in a well-established hind limb ischemia model. In the model, no tumors were observed. Mice transplanted with these cells displayed rapid recovery of limb blood perfusion, which was accompanied by a rapid increase in VEGF mRNA levels in the ischemic tissues.

Recently, preclinical studies had validated the concept of "cell therapy for ischemic disease" using stem/progenitor cells to repair and regenerate vascular cells in patients with severe ischemic cardiovascular disease [4,17]. However, collection of somatic stem cells in sufficient numbers for cell therapy is difficult and may require invasive procedures such as bone marrow aspiration or resection of the liver or pancreas. Additionally, problems of utilizing somatic stem cell is that (1) the number of somatic stem cell is very limited in general, (2) the implanted progenitor cells have low functions of mobilization capacity from bone marrow as well as angiogenic potentials in patients with multiple risk factors for arteriosclerosis [7-9,18]. On the other hand, iPS cells have an unlimited ability for self-renewal and expansion [10,19]. Thus, it might be possible to collect large numbers of cells following cultivation in vitro while maintaining its undifferentiated phenotype. iPS cells can also be differentiated into various kinds of cells as needed [11-16]. In addition, autologous iPS cells can circumvent the ethical controversy associated with ES cell and reduce immune-related problems [20]. Thus, iPS cells may have superiority for vascular regenerative medicine compared to ES cells and somatic stem/progenitor cells.

Therapeutic angiogenesis is likely mediated by multiple angiogenic cytokines released from implanted cells and host muscle tissues such as VEGF, bFGF and HGF rather than by direct differentiation of transplanted cells into mature endothelial cells [21-23]. Consistent with this notion, our study showed that implanted Flk-1^+ ^cells generated from iPS cells rapidly augmented expression of VEGF in ischemic tissues. On the other hand, the expression of bFGF, HGF or IGF was not affected in this study. Enhanced VEGF activity would directly affect hind limb blood flow.

Recently, Narazaki and coworkers showed that Flk-1^+ ^cells derived from mouse iPS cells could differentiate into both endothelial and mural cells and reproduce the vascular regeneration process [11]. In the present study, we confirmed incorporation of Flk-1^+ ^cells generated from iPS cells into vascular network structures in vitro. In addition, it was shown that implanted Flk-1^+ ^cells were successfully incorporated into capillary networks in the ischemic tissues. Therefore, Flk-1^+ ^cells generated from iPS cells might stimulate angiogenesis not only by a paracrine action of cytokines but also by a direct side supply of endothelial cells. These findings are encouraging since it is possible that implantation of iPS cell-derived Flk-1+ cells could construct de novo vessels in ischemic tissues, consistent with postnatal vasculogenesis. Further studies will be needed to clarify the differentiation capacity of these cells.

Recently, two studies reported the usefulness of Flk-1^+ ^cells for promoting angiogenesis in vivo [24,25]. Transplantation of human cord blood-derived Flk-1^+^/CD34^+ ^cells could salvage ischemic tissue in severe combined immune deficiency mice [24]. When Flk-1^+ ^cells generated from ES cells were directly injected into the heart, significant improvement in cardiac function was observed in doxorubicin induced cardiomyopathy, and this was accompanied by an increase in capillary density [25].

We have some limitations in this study. Firstly with regard to the unexpected lack of difference in VEGF expression in day 14, self-healing and supplementary effect by iPS may spontaneously attenuate around 14 days after transplantation. One study showed that double immunolabeling for BrdU and CD-31 in ischemic versus normal murine hindlimbs established that endothelial cell proliferation peaks at 7 d (1,235 ± 254 versus 8 ± 14 BrdU-positive cells/mm^2 ^for the ischemic versus normal limbs, respectively, P < 0.001); proliferative activity is then subsequently reduced at days 14 and 21[26]. However, detailed biochemical and cellular studies are required to better understand the underlying mechanism of these conditions.

Secondly, the KSN nude mice were used for this study to avoid immunological rejection of the injected iPS cells, because the genetic background of the MEFs for iPS generation in this study was 75% DBA, 12.5% C57BL/6 and 12.5% 129S4 [27]. KSN nude mice were mostly dead within three month by intestinal infections. We could not detect formation of any tumors in the mice transplanted Flk-1^+ ^cells at least until post-operative day 60. Because more than 99% of MACS-sorted cells were Flk1^+^, the contamination of undifferentiated cells into injected cells was very rare. The present study indicated the possibility of therapeutic use of iPS cells. However, we always need very careful observation for tumor formation in any transplantation studies using iPS cells in the future.

## Conclusion

In conclusion, direct local implantation of mouse iPS cell-derived Flk-1^+ ^cells has augmented ischemia-induced angiogenesis in a mouse model. This suggests that iPS cells would be a potential candidate for use in therapeutic angiogenesis.

## Methods

### Reagents

Allophycocyanin (APC) conjugated anti-mouse Flk-1, Phycoerythrin (PE) conjugated anti-mouse Sca-1 and PE-CD44 antibodies were purchased from eBioscience (San Diego, California). Anti-APC microbeads were purchased from Miltenyi Biotec (Bergisch Gladbach, Germany). PE conjugated anti-mouse CD11b, PE-CD45, PE-CD117(c-kit), PE-CD31, PE-CXCR4, Fluorescein isothiocyanate (FITC) conjugated anti-mouse CD34, FITC-CD90 and CD31 monoclonal antibodies were purchased from BD Pharmingen (San Diego, California). PE-SSEA1 antibody was purchased from R&D Systems (Minneapolis, Minnesota). PE-VE-cadherin antibody was purchased from Santa Cruz Biotechnology (Santa Cruz, California). Alexa Fluor^® ^555 conjugated E-Cadherin antibody was purchased from Cell Signaling Technology (Danvers, Massachusetts). PKH26 Red Fluorescent Cell Linker Kit was purchased from SIGMA-ALDRICH (St Louis, Missouri).

### Cell Culture

Germline competent mouse iPS cell lines "iPS-MEF-Ng-20D-17" generated from mouse embryonic fibroblasts by introducing the four factors(Oct3/4, Sox2, Klf4 and the c-Myc mutant c-Myc(T58A) with the use of retroviral vectors were provided by Riken Cell Bank with the permission of Dr. S. Yamanaka [10,27]. iPS-MEF-Ng-20D-17 cell is carrying Nanog promoter-driven GFP/IRES/puromycin-resistant gene (Nanog-iPS cells). iPS cells were maintained in Dulbecco's modified Eagle's medium (Invitrogen, Van Allen Way Carlsbad, California) containing 10% Knockout Serum Replacement (KSR)(Invitrogen), 1% fetal bovine serum (FBS), nonessential amino acids, 5.5 mmol/L 2-mercaptoethanol, 50 U/mL penicillin, and 50 mg/mL streptomycin on feeder layers of mytomycin-C-treated mouse embryonic fibroblast cells stably releasing leukemia inhibitory factor (LIF). Cell differentiation was induced as described previously[11]. In brief, differentiation medium (DM) (α-minimum essential medium (Invitrogen) supplemented with 10% FBS and 5 × 10^-5 ^mol/L 2-mercaptoethanol) was used for iPS cell differentiation. Flk-1^+ ^mesodermal cells were induced by 96 to 108 hr culture of iPS cells (plated at 1.7 × 10^3 ^cells/cm^2^) in DM in the absence of LIF on type IV collagen-coated dishes (ASAHI GLASS CO., LTD, Tokyo, Japan).

### Cell separation and analysis

Cultured cells were harvested after induction of Flk-1^+ ^cells by 96 to 108 hr culture in DM on type IV collagen-coated dishes. Induced cells were stained with APC conjugated anti-mouse Flk-1 antibody. Flk-1^+ ^cells were sorted with a magnetic cell separation system and purity was confirmed by flow cytometric analysis (BD FACS Canto, BD, Franklin Lakes, New Jersey). For the detailed characterization, induced cells were stained with APC conjugated anti-mouse Flk-1 antibody and additional antibody (CD11b, CD44, CD45, CD117, Sca-1, SSEA-1, E-cadherin, CXCR4, CD31, VE-cadherin, CD34 and CD90). Stained cells were analyzed by BD FACS Canto.

### Tube formation assay and incorporation of Flk-1^+ ^iPS cells

The formation of vascular-like structures by Flk-1^+ ^iPS cells on growth factor-reduced Matrigel (BD Biosciences, Bedford, Massachusetts) was performed as described previously[28]. Briefly, iPS positive cells (labeled with the PKH26 Red Fluorescent Cell Linker Kit [SIGMA-ALDRICH, St Louis, Missouri]) and HUVEC(Cambrex Bio Science Walkersville, Inc., Charles City, Iowa) were seeded at a ratio of 1:1 on coated plates at 3 × 10^4 ^cells/cm^2 ^in EBM-2 medium containing EGM-2(LONZA, Basel, Switzerland) and incubated at 37°C for 24 h. Network formation and Flk-1^+ ^iPS cell incorporation were assessed using an inverted phase contrast microscope (Biozero BZ8000, KEYENCE Japan, Osaka, Japan).

### Mouse model of hind limb ischemia and cell transplantation

Male KSN athymic nude mice were used for this study. Study protocols were approved by the Institutional Animal Care and Use Committee (IACUC) of Nagoya University School of Medicine. Mice, ages 8 to 12 weeks, were subjected to operative unilateral hind limb ischemia under anesthesia with sodium pentobarbital (50 mg/kg i.p.). In this model, the entire left femoral artery and vein were excised surgically [29]. Before surgery and on postoperative days three, seven, 14, and 21, body weight and systolic blood pressure were determined using a tail-cuff pressure analysis system in the conscious state. Flk-1^+ ^cells (2 × 10^3^, 2 × 10^4 ^or 6 × 10^4 ^cells/mouse) or PBS as a control were injected into four different sites of adductor muscles in the ischemic limb on postoperative day one. To trace transplanted cells in the ischemic tissues, sorted Flk1^+ ^cells were labeled with a PKH26 Red Fluorescent Cell Linker Kit and then injected into ischemic adductor muscles. Implanted cells were evaluated by immunohistochemical analysis 21 days after cells implantation. The signals were detected and analyzed by fluorescence microscopy.

### Laser Doppler blood flow analysis

Hindlimb blood flow was measured using a laser Doppler blood flow (LDBF) analyzer (Moor LDI; Moor Instruments, Devon, United Kingdom). LDBF analysis was performed on legs and feet. Blood flow was displayed as changes in the laser frequency using different color pixels. After scanning, stored images were analyzed to quantify blood flow. To avoid data variations due to ambient light and temperature, hindlimb blood flow was expressed as the ratio of left (ischemic) to right (nonischemic) LDBF in a same mouse according to previous studies [26,29-33]. In addition, in this study, the each ratio of left (ischemic) to right (nonischemic) LDBF was normalized to the each day-1(pre surgery) LDBF value.

### Reverse transcriptase-polymerase chain reaction and real-time Reverse transcriptase-polymerase chain reaction

Total RNA was isolated from cultured iPS cells using TRIzol Reagent (Invitrogen Life Technologies, Carlsbad, CA). Total RNA from ischemic muscles was extracted using the of FastRNA Pro Green Kit. (MP Biomedicals, Solon, Ohio). The cDNA was produced using oligo-dT primers and superscript II reverse transcriptase (superscript II, Invitrogen). The cDNA was diluted with DNase-free water at a concentration of 10 ng/μl. RT-PCR was performed using the Ex-Taq PCR kit (Takara, Otsu, Japan) according to the manufacturer's instructions.

Real-time reverse transcriptase-polymerase chain reaction (real-time RT-PCR) was performed using 1 μg cDNA in the Mx3000P Real-Time PCR System (Stratagene, Agilent Technologies, Santa Clara, California) using SYBR Green I as a double-stranded DNA-specific dye according to the manufacturer's instructions (Applied Biosystem, Foster City, California).

Primers were as follows: forward 5'-CAGGCTGCTGTAACGATGAA-3' (Location: Exon3, Melting Temperature:60.01°C) and reverse 5'-GCATTCACATCTGCTGTGCT-3' (Location: Exon4, Melting Temperature:60.02°C), Product size: 140 bp for murine VEGF.; forward 5'-GGCGGTGGTGACAGTATCTT-3' (Location: Exon3, Melting Temperature:60.00°C) and reverse 5'-GTCACTGACAGAGGCGATGA-3' (Location: Exon4, Melting Temperature:59.99°C), Product size: 162 bp for murine Flk-1.;forward 5'-CCAATCAGCTTGGGCTAGAG-3' (Location: Exon5, Melting Temperature:59.97°C) and reverse 5'-CTGGGAAAGGTGTCCCTGTA-3' (Location: Exon6, Melting Temperature:59.96°C), Product size: 129 bp for murine Oct3/4.;forward 5'-AAGTACCTCAGCCTCCAGCA-3' (Location: Exon3, Melting Temperature:60.01°C) and reverse 5'-GGGGATAGCTGCAATGGATG-3' (Location: Exon5, Melting Temperature:62.66°C), Product size: 199 bp for murine Nanog.;forward 5'-TCCTGTACCTCGTCCGATTC-3' (Location: Exon2, Melting Temperature:60.07°C) and reverse 5'-GGTTTGCCTCTTCTCCACAG-3' (Location: Exon3, Melting Temperature:59.84°C), Product size: 195 bp for murine c-myc.;forward 5'-AGTGTGACGTTGACATCCGT-3' (Location: Exon5, Melting Temperature:59.02°C) and reverse 5'-GCAGCTCAGTAACAGTCCGC-3' (Location: Exon7, Melting Temperature:61.15°C), Product size: 298 bp for murine beta actin.; forward 5'-AACTTTGGCATTGTGGAAGG-3' (Location: Exon3, Melting Temperature:59.97°C), and reverse 5'-ACACATTGGGGGTAGGAACA-3'(Location: Exon3, Melting Temperature:60.09°C), Product size: 223 bp for murine GAPDH.; forward 5'-AGCGGCTCTACTGCAAGAAC-3'(Location: Exon1, Melting Temperature:59.79°C), and reverse 5'-GCCGTCCATCTTCCTTCATA-3'(Location: Exon4, Melting Temperature:60.04°C), Product size: 183 bp for murine FGF2.; forward 5'-GGCAGCTATAAAGGGACGGTA-3' (Location: Exon5, Melting Temperature:60.46°C), and reverse 5'-CTTCTTCCCCTCGAGGATTT-3'(Location: Exon6, Melting Temperature:59.65°C), Product size: 154 bp for murine HGF.

Analyses of mRNA levels of VEGF were normalized to GAPDH as the internal control and expressed relative to the quantity of VEGF mRNA at day three in ischemic adductor muscle injected with PBS (day three control).

### Statistical Analysis

All data were obtained from at least three independent experiments. Statistical analysis of the data was performed using Student's t test or two-way ANOVA. P < 0.05 was considered significant. All data are shown as means ± SEM.

## List of abbreviations

iPS cell: induced pluripotent stem cell; PAD: peripheral arterial disease; Flk-1: fetal liver kinase 1; ES cell: embryonic stem cell; VEGF: vascular endothelial growth factor; BM: bone marrow; EPCs: endothelial progenitor cells; LDBF: laser doppler blood flow; HUVEC: human umbilical vein endothelial cell

## Competing interests

The authors declare that they have no competing interests.

## Authors' contributions

HS carried out the all experiments, participated in the analysis and interpretation of data and drafted the manuscript. TK carried out flow cytometry analysis and in vivo experiments. sequence alignment and drafted the manuscript. MK, PL and TY carried out in vitro differentiation and in vivo analysis. NN, SI and YN participated in the design of the study and the analysis and interpretation of data. JY participated in the design of the study and advised to handling the stem cell and differential methods. RS, TM and KI conceived of the study, and participated in its design and coordination and helped to draft the manuscript. All authors read and approved the final manuscript.
